# Are chatbots reliable text annotators? Sometimes

**DOI:** 10.1093/pnasnexus/pgaf069

**Published:** 2025-04-01

**Authors:** Ross Deans Kristensen-McLachlan, Miceal Canavan, Marton Kárdos, Mia Jacobsen, Lene Aarøe

**Affiliations:** Department of Linguistics, Cognitive Science, and Semiotics, Aarhus University, Aarhus 8000, Denmark; Center for Humanities Computing, Aarhus University, Aarhus 8000, Denmark; Department of Political Science, Aarhus University, Aarhus 8000, Denmark; Center for Humanities Computing, Aarhus University, Aarhus 8000, Denmark; Center for Humanities Computing, Aarhus University, Aarhus 8000, Denmark; Department of Political Science, Aarhus University, Aarhus 8000, Denmark

**Keywords:** large language models, data annotation, social sciences, Open Science, Natural Language Processing

## Abstract

Recent research highlights the significant potential of ChatGPT for text annotation in social science research. However, ChatGPT is a closed-source product, which has major drawbacks with regards to transparency, reproducibility, cost, and data protection. Recent advances in open-source (OS) large language models (LLMs) offer an alternative without these drawbacks. Thus, it is important to evaluate the performance of OS LLMs relative to ChatGPT and standard approaches to supervised machine learning classification. We conduct a systematic comparative evaluation of the performance of a range of OS LLMs alongside ChatGPT, using both zero- and few-shot learning as well as generic and custom prompts, with results compared with supervised classification models. Using a new dataset of tweets from US news media and focusing on simple binary text annotation tasks, we find significant variation in the performance of ChatGPT and OS models across the tasks and that the supervised classifier using DistilBERT generally outperforms both. Given the unreliable performance of ChatGPT and the significant challenges it poses to Open Science, we advise caution when using ChatGPT for substantive text annotation tasks.

## Introduction

The rapid development of large language models (LLMs), such as ChatGPT, has generated substantial scientific interest. Proponents suggest they show promise in solving research challenges, such as large-scale text annotation (1–3). However, this still nascent technology has significant drawbacks, especially with regards to Open Science. It is therefore important to evaluate their performance to: (i) advance understanding of their accuracy and reliability of measurement; (ii) clarify to what extent researchers face a trade-off between classification accuracy and Open Science principles; and (iii) stimulate discussion about whether such a trade-off is desirable or acceptable. In this paper, we systematically evaluate the performance of ChatGPT against alternative open-source (OS) models and supervised classifiers developed on a new set of data and tasks.

Early experimentation has highlighted the potential of ChatGPT for text annotation (1–3), but the empirical evidence is still limited, and other studies are more circumspect about the accuracy and reliability (4, 5). A preliminary review concluded that “evidence about their effectiveness remains partial” (4 p.1). Furthermore, ChatGPT is a closed-source, proprietary model, which raises significant Open Science concerns with respect to transparency and reproducibility, alongside cost barriers and data protection issues (4, 6).

These limitations can be overcome with both supervised classifiers and OS LLMs. These approaches make the underlying model openly available, can be used free of charge, and run on local hardware to mitigate data privacy and protection issues. Furthermore, recent research suggests that OS LLMs may “represent a competitive alternative for text annotation tasks, exhibiting performance metrics that generally exceed those of MTurk and rival those of ChatGPT” (7 p.6). Other research, however, finds that the current generation of LLMs, including both OS models and ChatGPT can be unreliable and unpredictable (5). While some technical solutions have been proposed as a remedy for these problems (8), there has been limited side-by-side comparison of these three potential solutions (i.e. OS LLMs, closed-source LLMs, and supervised classifiers) on novel data and tasks. Such a comparison is important because it allows for a direct comparison of classification performance.

We systematically evaluate the performance of contemporary OS LLMs and multiple ChatGPT models, with two additional supervised machine learning classifiers trained by the authors on human-labeled data. We evaluate model performance on two different binary classification tasks on a new dataset of tweets^a^ from US news media (*n* = 1,000 tweets for each task), experimenting with few- and zero-shot learning and using generic and custom prompts. To increase ecological validity, we focus on binary tasks because they have broad applicability across the social sciences and related fields (see “Materials and methods” for model architectures, data, and tasks). We choose not to fine-tune the LLMs since this can be technically challenging, requiring resources that are unavailable for most researchers. Our aim is to achieve a common ecological validity, focusing on how this technology is most likely to be used “in the wild.”

Overall, our findings suggest that researchers do not face a trade-off between classification accuracy and Open Science principles, since both OS and closed-source LLMs tend to underperform relative to certain supervised machine learning approaches, irrespective of model or prompting procedure. ChatGPT generally outperforms OS models, but we find significant inconsistency and unreliability across different tasks and models. Given the closed-source nature of ChatGPT, it is challenging to pinpoint the cause of this inconsistency. We conclude with recommendations for researchers on using LLMs for text annotation.

## Results

In our evaluations, we report both accuracy and F1. Accuracy, meaning the percentage of correct agreement relative to ground truth, is widely used and intuitive to interpret but is typically inflated when classes are unbalanced. Conversely, F1 is a more robust performance evaluation metric for unbalanced data, representing the harmonic mean of precision and recall. Full results of all models and classification reports can be found in the associated Harvard Dataverse repository.

**Fig. 1.**
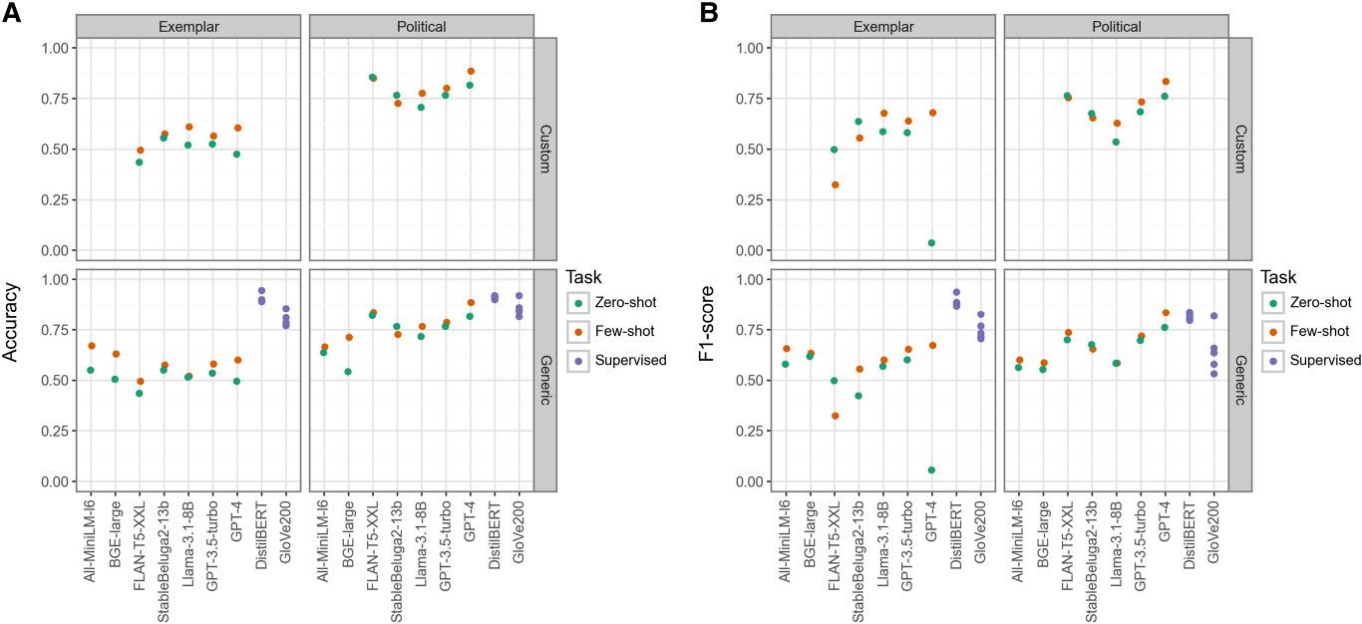
A) Accuracy (i.e. percentage of correct predictions) and B) F1 scores by model type, prompt type, and zero or few shot.

Figure 1A shows the accuracy, and Fig. 1B shows the F1 score of all models across all permutations. In each panel, the two columns represent the individual binary classification tasks, and the rows represent models with custom and generic prompts, respectively. The categories are the presence of political/nonpolitical and exemplar/nonexemplar content in the US news media tweets.

The supervised classifier using DistilBERT has the highest performance of all the models on both tasks, matched only by GPT-4 for predicting political content. No other model comes close to DistilBERT's performance for the exemplar task. FLAN-T5-XXL performs well in zero- and few-shot contexts on political labels with both custom and generic prompting. However, the same model performs worst for predicting exemplar label across all permutations.

The supervised model trained with static GloVe embeddings performed notably worse on predicting political label. Moreover, performance is generally worse on the exemplar task for all models regardless of prompt structure or zero versus few shots, learning with extreme variation in performance on this task. Perhaps most notably, GPT-4 performs significantly *worse* than any other model in the context of zero-shot labeling on exemplar data.

Our results show that, for both tasks, only the supervised DistilBERT model has consistently and meaningfully high enough performance above chance to be considered useful. Moreover, custom prompts seem generally to generate marginally higher performance than generic prompts for both ChatGPT and OS models for the exemplar task. However, most importantly, LLM performance on both tasks varies in unpredictable and inconsistent ways depending on the prompt and zero- or few-shot approach.

**Fig. 2.**
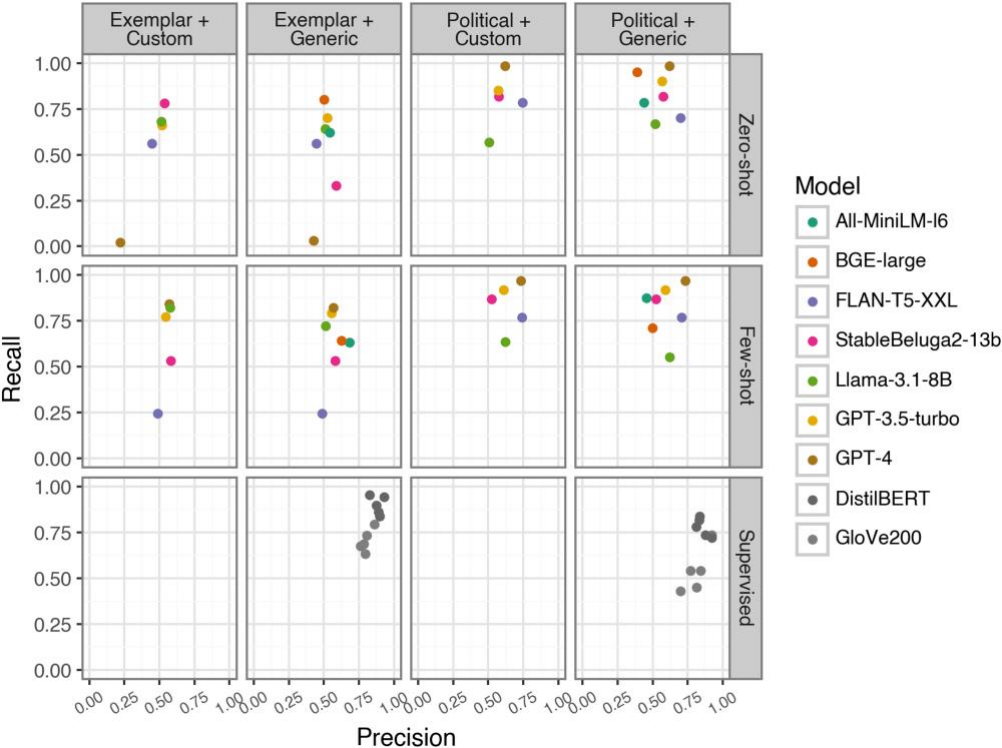
Precision and recall score of the positive class by model type and prompt.

Figure 2 expands on these results, reporting the recall and precision scores for the positive classes (i.e. “political” and “exemplar”). These results further support that the supervised classifiers mostly have a higher performance. Furthermore, the performance of the ChatGPT and OS models clusters around the same good-to-high performance levels for the political class with relatively few percentage-point deviance. However, for the exemplar class, all LLMs have equally low precision, with greater variation in recall. Most strikingly, GPT-4 demonstrates recall as low as 0.1 when classifying exemplar tweets (zero-shot, generic prompt).

The intercoder reliability scores for the multicoded data indicate strong intercoder reliability among the human coders: *α* = 0.86 (political) and 0.84 (exemplar). Since humans did not reach perfect agreement, it may be unrealistic to expect tools for automated classification to reach perfect accuracy. Nevertheless, the variation in Fig. 1 suggests that the performance of LLMs on particular tasks is not easily predictable from human performance.

These results are significant for a number of reasons. First, although the performance of the LLMs is impressive in places, the supervised machine learning approach—especially DistilBERT—generally performs best. Second, while GPT-4 in particular marginally outperforms OS LLMs, in other instances, the OS models are within a few percentage points of this model or even outperform it. Additional prompt engineering may improve performance, and testing this iteratively is significantly simpler and cheaper when using a smaller model like Llama3.1-8b which can be run locally, rather than paying on a token-by-token basis for API access to ChatGPT.

## Discussion

The performance of ChatGPT and other OS LLMs varies significantly and often unpredictably on our binary classification task. The different OpenAI models in particular demonstrate significant variation, and the source of this is opaque, since OpenAI's closed-source policies make testing and evaluation impossible. Closed models slightly outperform OS LLMs on certain tasks, but both are consistently matched or outperformed by the supervised classifier using DistilBERT. These results are more cautious about the potential of ChatGPT than earlier studies (1, 2, 7) and align with more cautionary conclusions (4, 5).

Furthermore, OpenAI (and similar) continuously adjust their models, meaning that research and annotation conducted using ChatGPT today may not be directly replicable or even reproducible in 6 months. Our findings indicate that these challenges to Open Science come with limited performance gains compared with OS alternatives and typically none compared with the supervised classifier. On this basis, we advise caution in using ChatGPT for text annotation in social science research at this time and conclude that the most consistent way for researchers to perform reliable, transparent, and efficient text annotation at scale is through human labeling of data and training supervised classifiers.

A limitation of our study is that our results are based on a single dataset in English, meaning generalizability may be limited. Recent research finds that ChatGPT performs similarly or (slightly worse) in other languages (3) and other types of text data (1). Our analysis also does not include fine-tuning of LLMs, which may improve performance. We find that custom prompting and few-shot examples generally improve performance, but the effects are inconsistent, and further research is needed.

While Generative AI continues to capture both popular imagination and the attention of researchers, our results demonstrate that LLMs are currently too brittle and potentially unreliable for scientific research. Moreover, closed-source versions are opaque and prohibitively expensive when working with large datasets. We argue that research time and finances are instead better invested in the development and application of encoder-only models, such as DistilBERT, which can be integrated into standard supervised learning pipelines. While perhaps less immediately glamorous, these models continue to match or outperform decoder-only models for a fraction of the cost and with the added bonus of greater transparency and reproducibility.

## Materials and methods

### Data collection and annotation

We use a dataset of tweets from the US news media outlets collected from 2023 by the authors for a new research project. These tweets were annotated for whether or not they contained political content and whether or not they contained an exemplar. All tweets were annotated by three research assistants. Any discrepancies were subsequently resolved by an expert coder (an author of this paper). On the multicoded data, the intercoder reliability between three research assistants was high (0.86 for political and 0.84 for exemplar); therefore, there was a clear shared understanding even before discrepancies were resolved. For each task, we have 1,000 multicoded tweets. This constitutes the ground truth data that was used to train the supervised classifier and to assess performance. From each of these 1,000 multicoded datasets, a fixed sample of 200 tweets was drawn for testing the LLM-based models.

### Supervised machine learning classifiers

Human-coded tweets were first transformed into dense numerical representations known as word embeddings. We train models using both static (GloVe) and contextual word embeddings (DistilBERT) (9, 10). These classifiers were trained using stratified *k*-fold cross-validation (*k* = 5).

### Large language models

We test a range of possible zero- and few-shot learning models with basic prompting. The models were selected based on (i) past classification performance, (ii) widespread usage at the time of writing, and (iii) accessibility (see details on prompt instructions in the Supplementary material). First, we use two-sentence transformers (11) which had demonstrated high levels of performance across a number of Natural Language Processing (NLP) tasks at the time of the initiation of the research: *all-minilm-L6* and *bge-largeen* (12). Second, for OS LLMs, we use *FLAN-T5-XXL* (13), a Text-to-Text model released by Google based on their earlier T5 architecture, which has shown promising classification performance (7). Third, we included autoregressive, decoder-only transformer models Llama-3.1-8B created by Meta and StableBeluga2-13b, a fine-tune of an earlier Llama2 model created by StabilityAI. Finally, we test two LLMs underlying ChatGPT, namely GPT-3.5-turbo and GPT-4, accessed via the OpenAI API.

## Supplementary Material

pgaf069_Supplementary_Data

## Data Availability

The data that support the findings of this study are openly available in Harvard Dataverse at https://doi.org/10.7910/DVN/TM7ZKD. Data are shared in accordance with Twitter/X's Developer Agreement, meaning that only post IDs can be shared, not the posts themselves. All Python code to reproduce our results can be available on Github and Zenodo at https://zenodo.org/records/14916594.

## Footnotes

a Twitter was rebranded to X in July 2023. It is unclear whether content produced on the platform should still be called “tweets” or whether an alternative term such as “posts” or “x’s” is preferred. For the sake of simplicity and continuity across our data, we adopt the earlier term “tweets” throughout this report.

## Preprints

This manuscript was posted as a preprint on arXiv: https://doi.org/10.48550/arXiv.2311.05769.
