# Supplementary material for: Are chatbots reliable text annotators? Sometimes
Source: PNAS Nexus. 2025 Apr 1;4(4):pgaf069. doi: 10.1093/pnasnexus/pgaf069 (PMC11954583; doi:10.1093/pnasnexus/pgaf069)
Supplement: pgaf069_Supplementary_Data [file pgaf069_supplementary_data.zip › PNASNEXUS-PNASNEXUS-2024-00415RR-s02.pdf]

S1. Codebook and Human Annotation

US News Media Tweet Dataset

Using the Twitter API we scraped tweets from approximately 360 American news media organizations from January 9, 2021, to January 4, 2023, for a new research project on political news communication on Twitter. In this report we analyze a random sample of 2,000 multi-coded tweets. The sample size of 2,000 tweets (1,000 for each code) was determined to ensure a reasonable number of tweets for training supervised machine learning classifiers with our cross validation strategy.

Annotation

Using a codebook, three student research assistants were trained to label tweets for the political and exemplar tasks (see codebook guidelines below). These tasks were chosen specifically since one is a broadly understood concept with a shared meaning, whilst the other is a more multivalent concept used infrequently in everyday language. All tweets were labeled by three research assistants where discrepancies were resolved by an expert coder (a co-author of this article). In the data annotated by three research assistants, there was high intercoder reliability between annotators - 0.86 for political and 0.84 for exemplar before the remaining discrepancies were resolved. These 2,000 tweets (1,000 for each code) constitute our gold standard for the political and exemplar task. This approach is in line with leading research on this topic - it minimizes false negatives/positives where the human label is wrong, and therefore gives a more accurate reflection of the performance of LLMs and supervised models.[3]

For the exemplar task, our data comprises 432 labeled as exemplar and a further 568 labeled as non-exemplar (approximately 1:1 ratio). For the political task, there are 247 tweets labeled as political and 753 as containing non-political (approximately 1:3 ratio).

Codebook Guidelines

We annotated for two tasks, political and exemplar. Below we report the definition for the political and exemplar task provided in the codebook. The codebook also included examples of tweets from each category and operational annotation instructions (code definition). The limited number of tweets concerning international news was labeled as ”non-exemplar” and ”non-political” as the broader research project for which the codebook was developed focuses on US domestic politics. The full codebook for the two tasks is available on the associated Dataverse for this report.

Political Task: Given we are focused on social media, the starting point for identifying political content builds on Twitter's definition of political content which it uses to regulate political content: '*Political content is defined as content that references a candidate, political party, elected or appointed government official, election, referendum, ballot measure, legislation, regulation, directive, or judicial outcome.*'

Another definition which can be used as a guide was developed by Petersen (2018, p.5)[4] for analysing Facebook posts by politicians: '*Political content includes 'all statements with a political substance, but excludes messages solely related to non-political matters such as birthdays or personal experiences*'. The message can express a political attitude directly or indirectly. For instance, a message saying "Thank you Emsworth Primary School for a nice visit" does not count as a political message, but if the message is followed with a political statement eg. "let's unite to secure good education for all" it should be coded as political. Further, political messages can comment on political processes and practices such as: "The government does not do what it takes to make the parties cooperate". Hence, a political message will

3. See for example Umansky, N., Kubli, M., Donnay, K., Gilardi, F., Hangartner, D., Kotarcic, A., ... and Grech, P. Enhancing Hate Speech Detection with Fine-Tuned Large Language Models Requires High-Quality Data. DOI:10.31219/osf.io/7kbqt

4. Petersen, H.H. (2018). Data Report: Facebook Posts of Danish MPs, https://ps.au.dk/fileadmin/ingen_mappe_valgt/Facebook_data_report_May_2018.pdf

generally state a policy position, raise awareness of a political cause, encourage people to take action to promote a political goal or reflect upon a political decision-making process.

Exemplar Task: Journalists often utilize examples of people to illuminate the story they are reporting on. Conceptually, these people are referred to as ‘exemplars’. For the purposes of this research, we adopt a broad definition of exemplars, as people who are mentioned in relation to the story being reported. They can be people directly affected and involved in the issue, or they may simply be commenting on the issue. This broader definition which includes unaffected and affected exemplars is illuminated in further detail by Zerback & Peter (2018).[5]

This definition expands on the classic understanding in the communication literature which required that the person portrayed be an active part of the story or directly affected. As a result, this expanded definition includes so-called ‘vox populi’ where people are simply giving their opinion on an issue or policy being reported.

S2. Model selection

As mentioned in the main text we test a range of possible zero- and few-shot learning models with basic prompting to evaluate the performance of LLMs. As mentioned in the main text, the models were selected based on 1) past classification performance and 2) widespread usage, and 3) accessibility. The OS LLMs included represent models released since late 2023 which have shown high-performance and wide user adoption. That the models tested include both examples from the beginning of this period of time and more recent OS LLMs increases the external validity.

As noted in the main text we do not fine-tune the LLMs on the task, since this is a technically challenging task requiring resources and which may therefore not be available to many scholars. Nevertheless, the degree of technical complexity of fine-tuning depends on the type of model, and recent research finds that fine-tuning can improve LLM performance and this is an important point for future research. [6]

S3. Few shot learning examples

In the few-shot approach, we provide the LLMs with some examples of tweets which are considered US politics and exemplars, and those which are not. These examples were randomly selected from tweets which all research assistant expert coders agreed represented the category in the training data. Random selection gives us breadth of coverage of different types of tweets in each category and using only tweets where all three research assistants agree gives us validity they capture the concept. The examples we provided were:

Political:

- ‘In my opinion, to be faithful is to tell both sides of the story.’ GOP Sen. Tim Scott discusses a part of his response to Biden’s address that almost didn’t happen and race relations in America. https://t.co/WbjnFJEibq
- A New Day: Why Neither Politicians Nor Clergy Can Hide From Abortion Any Longer https://t.co/jxFQKfTldr
- Finland and Sweden have mostly stopped prescribing blockers to under-18s in favour of talking therapy, because the evidence base for them is thin. Joe Biden’s order asks federal departments to expand access to “gender-affirming care” https://t.co/cmylNEJ0Gj

---

5. Zerback, T., & Peter, C. (2018). Exemplar effects on public opinion perception and attitudes: The moderating role of exemplar involvement. Human Communication Research, 44(2), 176-196.

6. See for example Umansky, N., Kubli, M., Donnay, K., Gilardi, F., Hangartner, D., Kotarcic, A., ... and Grech, P. Enhancing Hate Speech Detection with Fine-Tuned Large Language Models Requires High-Quality Data. DOI:10.31219/osf.io/7kbqt and Alizadeh, M., Kubli, M., Samei, Z., Dehghani, S., Bermeo, J. D., Korobeynikova, M., & Gilardi, F. (2024). Open-source large language models outperform crowd workers and approach ChatGPT in text-annotation tasks. arXiv preprint arXiv:2307.02179v2.

- ’We’re broken.’ In the suburbs north of Los Angeles, voters feel fed up and afraid https://t.co/Gzxc6zlhnS
- House Democrat Chris Pappas is in a close reelection race in New Hampshire. Good news: Sabato just upgraded the race from “Toss up” to “Lean Democrat.” But it’ll still be close. Donate right now and help put @ChrisPappasNH over the top: https://t.co/QjHgEpVXl4

Non-political (including international):

- Rialto police apologize to teenage girl’s family after violent arrest is caught on video https://t.co/xdZhIaKthC
- Following months during which Kanye West had been publicly addressing his divorce and co-parenting, he has reportedly told Kim Kardashian that he is ‘going away to get help.’ https://t.co/VbubgWmkdS
- Expect the return of holiday favorites, including the beloved classic ‘Santa Claus Is Comin’ to Town’ and a special airing of ‘Home Alone’ on Christmas Eve. https://t.co/w6kiFc8JDM
- Haaretz reports that according to some witnesses, the crowd celebrating Itamar Ben-Gvir’s election to the Israeli Knesset “chanted ‘death to Arabs’ alongside the more prevalent calls for ‘death to terrorists.’” https://t.co/9N4rwBybU7
- #ANALYSIS: ’Is your leadership safe?’: The question that shows how much Morrisons position has fallen https://t.co/ApJJLCc1JC

Exemplar:

- ‘The twists and turns in Spears’ story over recent years have fundamentally altered the dream of becoming a pop star, even as the appeal of finding one artist who can make a song that changes the world for five minutes remains’ writes @maura https://t.co/os1SH3Drsg
- RT @TedAbram1: YES!!! House Minority Leader Kevin McCarthy said Republicans are launching an investigation to find out why American tax dol[7]
- Two men rescued after 29 days lost at sea, survived on oranges and rainwater https://t.co/f5GGziG3vr https://t.co/RxlCNvJ4Xe
- MSNBC analyst takes up arms in Ukraine because he’s ’through talking about it’ https://t.co/5DvDO2780C
- RT @emilytgreen: “They left him as if he were—I won’t say an animal, because animals are sentient beings,” the family’s lawyer said. “As if…

Nonexemplar (including international):

- Operation Holiday Cheer sends Christmas trees to troops — https://t.co/EZaojUcXuX
- Border control advocates want Americans to snitch on ICE’s ‘secret night flights’ https://t.co/mMNXVmE7vg https://t.co/iJtgNlV1wa
- RT @MikeyNoWay: #Spinout on WB Hwy 24 near Wilder Rd. leaves right lane blocked by crews for overturned vehicle. A second crash tied up tra…
- The Healthy School Meals for All program is an investment in public education and in our children who need healthy food to learn. https://t.co/RL75NzNjx3
- https://t.co/7clmW8jHe3[8]

S4. Stormtrooper package

To perform comparable tests (both the original analysis and replication), we utilized the Python package stormtrooper (Kardos, 2023) [9] which is designed to perform zero- and few-shot learning with transformer-

7. Due to the scraping procedure for retweets, the final characters of the original tweet can be cut, we still code these tweets where there is enough information to indicate the code

8. Some of the tweets scraped in the dataset are URLs only. These were coded as not having an exemplars or political content present

9. Kardos, M. (2023). stormtrooper: scikit-learn compatible zero and few shot learning in Python, https://github.com/centre-for-humanities-computing/stormtrooper

based models. The package has a series of default prompts for this task, which can be found in the relevant modules in the open-source GitHub repo for the package. The full set of prompt instructions for this report can be found both in the associated Dataverse and on the Github repository for the stormtrooper package. [10]

For example, the default zero-shot prompt for generative decoder-only models such as StableBeluga-13B the generic prompt template is as follows:

### System:
You are a classification model that is really good at following instructions and produces brief answers that users can use as data right away.
Please follow the user's instructions as precisely as you can. **### User:**
Your task will be to classify a text document into one of the following classes:
{*CLASSES*}.
Please respond with a single label that you think fits the document best.
Classify the following piece of text:
{*INPUT_TEXT*}
### Assistant:

For a text-to-text model such as FLAN-T5-XXL, the generic prompt is much simpler:

I will give you a piece of text. Please classify it as one of these classes: classes.
Please only respond with the class label in the same format as provided.
{*INPUT_TEXT*}

S5. Prompting

The user of stormtrooper is free to design their own custom prompts which are more specifically targeted towards the task at hand through a process of prompt engineering. We hence also experimented with custom prompts which included definitions of the categories we with to annotate. A full overview of prompts is reported on Github. Examples of these custom prompts for StableBeluga-13B are as follows:

Zero-shot custom prompt:

### System:
You are a classification model that is really good at following instructions and produces brief answers that users can use as data right away.
Please follow the user's instructions as precisely as you can.
### User:
Your task will be to classify a text document into one of the following classes:
{*CLASSES*}.
Political content is defined as content that references a candidate, political party, elected or appointed government official, election, referendum, ballot measure, legislation, regulation, directive, or judicial outcome. Political content includes all statements with a political substance, but excludes messages solely related to non-political matters such as birthdays or personal experiences.
Please respond with a single label that you think fits the document best.
Classify the following piece of text:

10. Complete documentation for the stormtrooper package can also be found at the following URL: https://centre-for-humanities-computing.github.io/stormtrooper/

{*INPUT_TEXT*}
### Assistant:

Few-shot custom prompt:

### System:
You are a classification model that is really good at following instructions and produces brief answers that users can use as data right away.
Please follow the user's instructions as precisely as you can.
### User:
Your task will be to classify a text document into one of the following classes:
{*CLASSES*}.
Political content is defined as content that references a candidate, political party, elected or appointed government official, election, referendum, ballot measure, legislation, regulation, directive, or judicial outcome. Political content includes all statements with a political substance, but excludes messages solely related to non-political matters such as birthdays or personal experiences.
Here are some examples of texts labeled by experts. {*EXAMPLES*}
Please respond with a single label that you think fits the document best.
Classify the following piece of text:
{*INPUT_TEXT*}
### Assistant:

As before, the text-to-text models such as FLAN-T5-XXL use a simpler prompt set-up, as exemplified by the following custom few-shot prompt:

T5 few-shot custom prompt:
I will give you a piece of text.
Please classify it into one of the following classes: {*CLASSES*}.
Political content is defined as content that references a candidate, political party, elected or appointed government official, election, referendum, ballot measure, legislation, regulation, directive, or judicial outcome. Political content includes all statements with a political substance, but excludes messages solely related to non-political matters such as birthdays or personal experiences.
Here are some examples of texts labeled by experts.
{*EXAMPLES*}
Please only respond with the class label in the same format as provided. Label this piece of text:
{*INPUT_TEXT*}
